# Chloroplast Genome Sequence Annotation of <i>Dendrobium nobile</i> (Asparagales: Orchidaceae), an Endangered Medicinal Orchid from Northeast India

**DOI:** 10.1371/currents.tol.cf1709613759c2223eb582c0fa694cc7

**Published:** 2017-05-19

**Authors:** Devendra Biswal, Ruchishree Konhar, Manish Debnath, Sriram Parameswaran, Durai Sundar, Pramod Tandon

**Affiliations:** Bioinformatics Centre, North-Eastern Hill University, Shillong, Meghalaya, India; Bioinformatics Centre, North-Eastern Hill University, Shillong, Meghalaya, India; Bioinformatics Centre, North-Eastern Hill University, Shillong, Meghalaya, India; Genotypic Technology Pvt Ltd, Technology, Bengaluru, Karnataka, India; Biochemical Engineering and Biotechnology, Indian Institute of Technology Delhi, New Delhi, Delhi, India; Biotech Park, Lucknow, Uttar Pradesh, India

## Abstract

Orchidaceae constitutes one of the largest families of angiosperms. Owing to the significance of orchids in plant biology, market needs and current sustainable technology levels, basic research on the biology of orchids and their applications in the orchid industry is increasing. Although chloroplast (cp) genomes continue to be evolutionarily informative, there is very limited information available on orchid chloroplast genomes in public repositories. Here, we report the complete cp genome sequence of Dendrobium nobile from Northeast India (Orchidaceae, Asparagales), bearing the GenBank accession number KX377961, which will provide valuable information for future research on orchid genomics and evolution, as well as the medicinal value of orchids. Phylogenetic analyses using Bayesian methods recovered a monophyletic grouping of all Dendrobium species (D. nobile, D. huoshanense, D. officinale, D. pendulum, D. strongylanthum and D. chrysotoxum). The relationships recovered among the representative orchid species from the four subfamilies, i.e., Cypripedioideae, Epidendroideae, Orchidoideae and Vanilloideae, were consistent within the family Orchidaceae.

## Introduction

Chloroplasts are specialized intracellular organelles in which photosynthesis occurs, and they originated via an endosymbiotic relationship with cyanobacteria. Though most chloroplast genes are believed to have been transferred to the nucleus during evolution, their genomes have maintained fairly conserved structures and gene contents throughout their evolutionary lineage 1. While the complete plastid genomes of tobacco and liverworts were the first to be determined, as of April 2017, the complete chloroplast (cp) genomes of 1161 GenBank accessions from land plants have been reported in the National Centre for Biotechnology Information (NCBI) Organelle Genome Resources (http://www.ncbi.nlm.nih.gov/genome/browse/?report=5)^2^. Typically, the cp genomic size of land plants varies between 120 and 220 kb, with a pair of inverted repeats (IRs) that separate the genome into a large single copy (LSC) region and a small single copy (SSC) region^3^. Variation in the size of cp genomes among plant lineages is generally observed in the mutable IR region. The cp genomes of land plants usually contain approximately 110–120 genes, which mostly participate in photosynthesis or gene expression^4^^,^^5^. Information regarding gene content, polycistronic transcription units, sequence insertion or deletion, transition or transversion, and nucleotide repeats may help resolve evolutionary relationships in the kingdom Plantae (Viridiplantae)^6^^,^^7^^,^^8^.

The uniparental inheritance and non-recombinant nature of cp genomes make them potentially useful tools for inferring evolutionary and ancient phylogenetic relationships. Additionally, cp DNA data are easily obtainable from bulk DNA extractions, as multiple copies of these genomes are present in each cell, and they exhibit considerable sequence and structural variations within and between plant species^9^. Several chloroplast markers have been harnessed for phylogenetic analyses and taxonomic systematizations^10^. Complete cp genome sequencing and annotation provides important sequence information about suitable plastid DNA markers for the classification of plant species.

The advent of high-throughput sequencing has recently facilitated rapid advancements in the field of chloroplast genomics. Previously, such studies were performed on isolated chloroplasts, in which the entire chloroplast genome was amplified by rolling circle amplification. Recent progress in next-generation sequencing (NGS) technologies has paved the way for faster and cheaper methods to sequence organellar genomes^11^^,^^12^. There are multiple NGS platforms available for organelle genome sequencing, for which the Illumina platform is widely used, as it emphasizes the use of rolling circle amplification products^12^. At the time of writing this manuscript, cp genomes from 66 orchid species have been reported, according to NCBI Organellar genome records (http://www.ncbi.nlm.nih.gov/genome?term=txid4747[Organism: exp]%20NOT%20genome[PROP]%20AND%20non_genome[filter]). In the subfamily Epidendroideae, the genus Dendrobium contains nearly 1200 species, and the cp DNA sequences of only six Dendrobium species have been determined and deposited in GenBank.

*Dendrobium nobile* Lindl. is one of the most widespread species within the genus *Dendrobium* and is among the best-known plants used in traditional Chinese medicinal. *D. nobile* is an epiphytic or lithophytic plant native to the Indian subcontinent (Northeast India (including Assam and Sikkim), Bangladesh, Nepal and Bhutan), southern China (including Tibet), and Indochina (Myanmar, Thailand, Laos and Vietnam). Various parts of the plant are widely used as analgesics, antipyretics, and tonics to nourish the stomach in traditional medicine. Denbinobin, a natural product isolated from *D. nobile*, has a unique phenanthrene quinone skeleton and displays antitumor and anti-inflammatory activities^13^.

*D. nobile* is listed in the Convention on International Trade in Endangered Species of Wild Fauna and Flora (CITES) Appendix II, indicating that it is vulnerable to extinction if proper measures are not taken to control anthropogenic activities. In light of the need to preserve native species and use their chloroplast genome information in various molecular biology studies, we determined the cp genome sequence of *D. nobile* (GenBank Accession number: KX377961) from India and report it for the first time.

Another unpublished *D. nobile* chloroplast genome from China bearing the accession number KT591465 is archived in GenBank. A comparative study between these two genomes and the genome of the closely related species *Dendrobium officinale* reveals many evolutionary hotspots in the plastid genome, which are very useful for developing molecular markers to distinguish other *Dendrobium* species. Comparative chloroplast genomic analysis will be very useful for marker-assisted commercial breeding programs, chloroplast genetic engineering and systematic biology studies of *Dendrobium* species within the family Orchidaceae.

## Methods

**Plant materials and DNA extraction**

*D. nobile* plant specimens were collected from the National Research Centre for Orchids at Gangtok, Sikkim (Northeast India). A voucher specimen was deposited at the Department of Botany, North-Eastern Hill University, Shillong, India. Young fresh leaves were taken from the orchids that were grown in a greenhouse. High molecular weight DNA was extracted using a modified CTAB buffer that was purified using a column (Qiagen, GmBH, Germany). The DNA quantity was assessed using a spectrophotometer (Nanodrop 2000, Thermo Fischer, USA), and the DNA integrity was assessed by gel electrophoresis (using 0.8% Agarose, Sigma, USA). The quality and quantity of the genomic DNA were assessed using agarose gel electrophoresis, a Nanodrop and Qubit detection methods.

**NGS Library preparation**

Both paired-end and mate-pair libraries were prepared. Approximately 4 µg of Qubit-quantified DNA was used for tagmentation. The tagmented sample was cleaned using AMPURE XP beads (Beckman Coulter #A63881) and subjected to strand displacement. The 2-5 kb and 8-13 kb strand-displaced samples were size-selected using gel electrophoresis and subjected to circularization overnight. The linear DNA was then digested using DNA exonuclease. The circularized DNA molecules were sheared using a Covaris microTUBE with the S220 system (Covaris, Inc., Woburn, MA, USA) to obtain fragments of 300 to 1000 bp. The sheared DNA was subjected to M280 Streptavidin beads (Thermo Fisher Scientific, Waltham, MA) containing biotinylated junction adapters for purification. End repair, A-tailing, and adapter ligations were performed on the bead-DNA complex. The adapter-ligated sample was amplified for 15 PCR cycles (denaturation at 98˚C for 30 sec, cycling (98˚C for 10 sec, 65˚C for 30 sec and 72˚C for 30 sec) and a final extension at 72˚C for 5 min) and cleaned up using AMPURE XP beads (Beckman Coulter #A63881). The prepared library was quantified using Qubit and validated for quality by running an aliquot on D1000 ScreenTape (Agilent). The libraries were amplified for 9-11 cycles according to the Nextflex protocol and were quantified and sequenced on an Illumina NextSeq500 (Illumina, USA).

**Data processing**

The data quality of the Illumina WGS raw reads (151 bp x 2) was assessed using the FastQC tool. The raw reads were pre-processed using Perl scripts for adapter clipping and low-quality filtering. Reference *Dendrobium* chloroplast genomes (*D. officinale*, Accession: KJ862886; *D. huoshanense*, Accession: NC_028430 and *D. strongylanthum*, Accession: NC_027691) were retrieved from the NCBI-GenBank database. Adapter-clipped and low-quality trimmed processed reads were aligned to *Dendrobium* cp genomes using the BWA-MEM algorithm^14^ with the default parameter settings. Aligned reads were extracted, and k-mer–based de-novo assembly was achieved using the SPAdes-3.6.0 program (k-mer used 21, 33, 55 and 77) with the default parameter setting. The quality of the assembled genome was assessed by read alignment and genome coverage calculations using Samtools and Bcftools^15^ (https://samtools.github.io/bcftools/bcftools.html).

**Genome annotation**

Protein-coding and ribosomal RNA genes were annotated using the Basic Local Alignment Search Tool (BLAST; BLASTN, PHI-BLAST and BLASTX)^16^, CGAP^17^ and DOGMA^18^. The boundaries of each annotated gene were manually determined by comparison with orthologous genes from other orchid cp genomes. The tRNA genes were predicted using ARAGORN^19^. The circular genome maps were drawn using OGDRAW, followed by manual modification^20^. The sequencing data and gene annotations were submitted to GenBank, and their accession number (KX377961) was acquired.

**Nucleotide sequence diversity and phylogenetic application of cp genomes in the family Orchidaceae**

Whole chloroplast genome datasets from plant species representing four subfamilies of Cypripedioideae, Epidendroideae, Orchidoideae and Vanilloideae in the family Orchidaceae were aligned, and a comparative genome rearrangement was separately drawn using MAUVE^21^ with default parameters. The combined matrix was utilized for the phylogenomic analyses. A Bayesian inference (BI) tree was constructed using two independent Metropolis-coupled Markov chain (MCMC) runs using MrBayes 3.2.6^22^. Two parallel Bayesian analyses with four chains each, partitioned by the DNA region, were run for 50 million generations. Trees were constructed using a general time reversible substitution model (GTR) with substitution rates estimated by MrBayes 3.2.6. Metropolis-coupled Markov chain Monte Carlo (MCMCMC) sampling was performed with two incrementally heated chains that were combinatorially run for 100,000 generations. Coalescence of the substitution rate and the rate model parameters were also examined. The average standard deviation of the split frequencies was calculated, and generations were added until the standard deviation value was below 0.01. Posterior probabilities indicated clade support (100%). A cladogram with the posterior probabilities for each clade and a phylogram with mean branch lengths were generated and subsequently examined using FigTree v1.3.1 (http://tree.bio.ed.ac.uk/software/figtree/). The phylogenetic groupings in the family Orchidaceae were colour-coded based on sub-family groupings.

## Results

The complete cp genome of *D. nobile* was determined from a whole genome project initiative of the same species using paired-end and mate-pair data from Illumina HiSeq with 150*2 and Illumina NextSeq500 with 75*2, respectively. Further, the aligned Illumina reads were separated and assembled using CLC Main Workbench version 7.7.1. The Indian monoisolate *D. nobile* chloroplast genome is circular; is 152,018 bp long; and has a 62.53% A + T content, and it is similar to the previously determined chloroplast DNA of *D. nobile* from China (accession number KT591465; 153,660 bp; 62.50%). A total of 134 unique genes were successfully annotated, including 79 protein-coding genes, 8 rRNA genes, 7 pseudogenes and 38 tRNA genes (Fig. 1). The cp genome also comprised LSC (1.84,944; 84,944 bp), SSC (111,230.125,733; 14,504 bp), and two IR regions of 26,285 bp: IRA (84,945.111,229) and IRB (125,734.152018). A total of 20 genes were duplicated in the IR, 81 genes in the LSC and 11 genes in the SSC regions of the genome. In total, there were 12 genes {rps16, atpF, rpoC1, ycf3, rps12 (2), clpP, petB, rpl2 (2), ndhB (2)} with introns.

**Figure:**
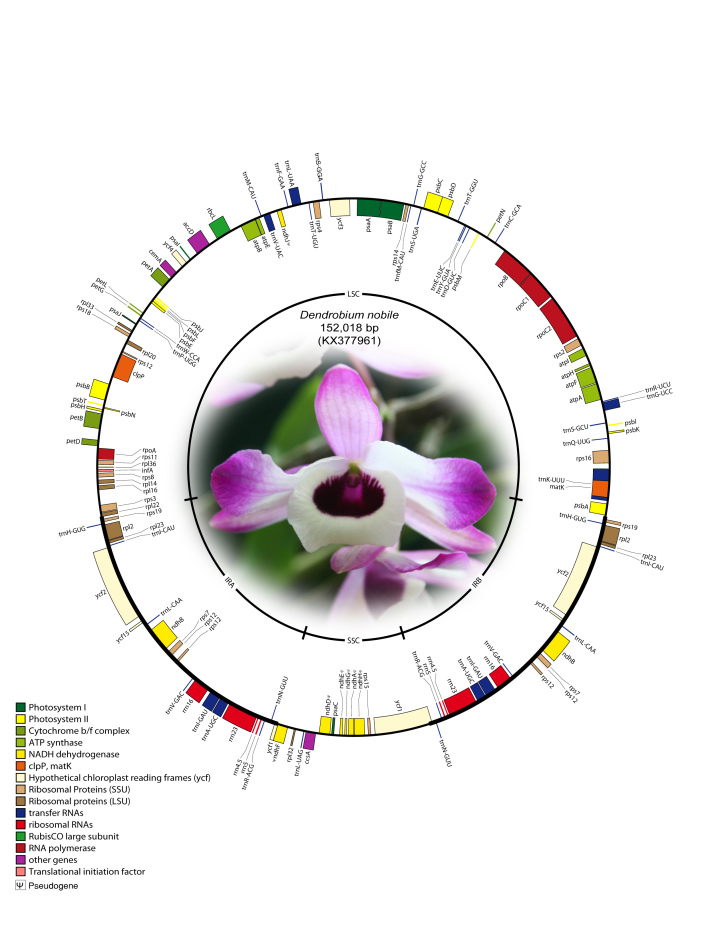
**Fig. 1: Annotated gene map of *Dendrobium nobile* chloroplast genome.** Genes shown inside the circle are transcribed clockwise, and genes shown outside the circle are transcribed counterclockwise. Genes belonging to different functional groups are colour-coded. A pair of inverted repeats (IRA and IRB) separates the genome into large single-copy (LSC) and small single-copy (SSC) regions in the inner circle; ψ indicates an ndh pseudogene.

Chloroplast sequences have been used in deep phylogenetic analyses because of their low substitution rates^23^. Complete chloroplast genomes have often been utilized to resolve relationships among angiosperms^24^. However, whole-genome sequencing using sparse sampling can result in long-branch artefacts and incorrect evolutionary reconstructions. Previous studies on the complete chloroplast genomes of *D. officinale* and six other orchid species have highlighted deep phylogenomic analyses based on their chloroplast genome organization^25^. Luo *et al*. (2014) achieved consistent results for the relationships among *Phalaenopsis* (Aeridinae), *Cymbidium* (Cymbidiinae), *Dendrobium* (Dendrobiinae), *Oncidium* and *Erycina* (Oncidiinae) within the subfamily Epidendroideae. Their analysis revealed structural similarities, but differences in IR/SSC junctions and ndh genes were also reported, which can be used as markers to identify species of orchids^25^.

In the present study, we sequenced the entire *D. nobile* cp genome from plant material collected from Northeast India. Twenty-three whole cp genome sequences spanning four subfamilies in the family Orchidaceae were also retrieved from GenBank.

A comparative whole genome rearrangement showing homologous alignment segments was drawn using all 23 known cp DNA sequences. Each genome is displayed horizontally, and homologous segments are shown as coloured blocks that are connected across genomes (Fig. 2A). Inverted segments in the genomes are represented by blocks with a downward shift relative to the reference genome. Sequence regions covered by a coloured block are entirely collinear and homologous among the genomes. The breakpoints of genome rearrangements are represented by boundaries of coloured blocks unless a sequence has been gained or lost in the breakpoint region. A Bayesian phylogenetic tree with 1000 bootstrap values was computed.

In our analysis of the relationships among the four subfamilies within the family Orchidaceae, all of the representative orchid species from each subfamily were well resolved into monophyletic clades. The analysis further exhibited a congruent monophyletic grouping of *Dendrobium* species (*D. nobile*, *D. huoshanense*, *D. officinale*, *D. pendulum*, *D. strongylanthum* and *D. chrysotoxum*) in the overall tree topology (Fig. 2B).

**Figure:**
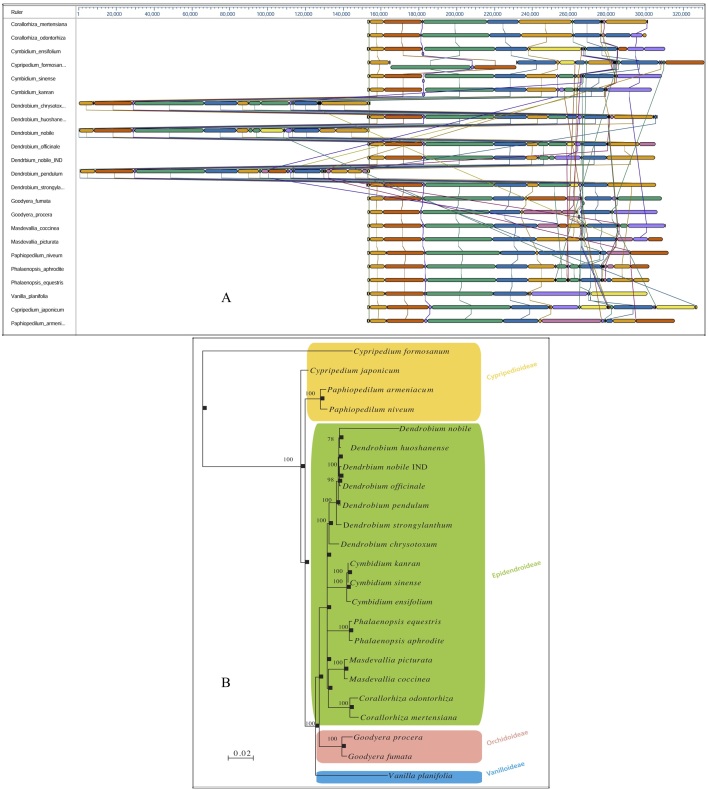
**Fig. 2: Bayesian phylogenetic tree of the family Orchidaceae, reconstructed based on whole chloroplast genomes** **A.** Whole chloroplast genome alignment of 23 orchid species representing four subfamilies, Cypripedioideae, Epidendroideae, Orchidoideae and Vanilloideae, in the family Orchidaceae. Each genome panel contains the name, the sequence coordinates for the genome, and a single black horizontal centre line with coloured block outlines appearing above and below. Each block is homologous and internally free of genomic rearrangement and is connected by lines to similarly coloured blocks depicting comparative homology across genomes. **B.** Phylogenetic trees from the whole genome alignment matrix yielded monophyletic groupings of the four orchid families. Posterior probability/bootstrap values are indicated near the nodes, which are quite supportive of the overall tree topology. The taxon-wise GenBank accession numbers for the published sequences are as follows: *Corallorhiza mertensiana* (NC_025661.1), *Corallorhiza odontorhiza* (NC_025664.1), *Cymbidium ensifolium* (NC_028525.1), *Cymbidium kanran* (NC_029711.1), *Cymbidium sinense* (NC_021430.1), *Cypripedium formosanum* (NC_026772.1), *Cypripedium japonicum* (NC_027227.1), *Dendrobium chrysotoxum* (NC_028549.1), *Dendrobium huoshanense* (NC_028430.1), *Dendrobium nobile* (NC_029456.1), *Dendrobium officinale* (NC_024019.1), *Dendrobium pendulum* (NC_029705.1), *Dendrobium strongylanthum* (NC_027691.1), *Dendrobium nobile* NE_India (KX377961), *Goodyera fumata* (NC_026773.1), *Goodyera procera* (NC_029363.1), *Masdevallia coccinea* (NC_026541.1), *Masdevallia picturata* (NC_026777.1), *Paphiopedilum armeniacum* (NC_026779.1), *Paphiopedilum niveum* (NC_026776.1), *Phalaenopsis aphrodite* (NC_007499.1), *Phalaenopsis equestris* (NC_017609.1), and *Vanilla planifolia* (NC_026778.1).

**Nucleotide sequence diversity and SSR analysis**

A detailed comparative account of the nucleotide sequence statistics are outlined in Tables 1-5. This descript includes atomic counts for single and double stranded DNA; nucleotide counts; A, T, G, C content and frequencies; codon usage and frequency; and nucleotide count in the codon positions of *D. nobile* genomes reported from Northeast India and China and of the reference genome *D. officinale*. Nucleotide sequence diversity, codon statistics from coding regions, AT/GC counts and simple sequence repeats (SSR) were computed for selected *Dendrobium* species using CLC Workbench 7.7.1. The complete *D. nobile* Indian isolate cp DNA (KX377961) is comprised of 15,2018 bases, whereas the Chinese isolate (LC011413) is 15,0793 bp in length. The nucleotide variation for A and T and the G-C percentage were higher in the Indian isolate than in the Chinese isolate. A total of 79 CDS, 22 exons, 132 genes, 2 repeat regions, 8 rRNAs and 38 tRNAs were reported from the cp DNA from the Indian isolate, whereas the Chinese isolate comprises 73 CDS, 127 genes, 2 repeat regions, 8 rRNAs and 39 tRNAs (Tables 1-5). In total, 46, 42, and 46 SSRs were identified for *D. officinale*, *D. huoshanense* and *D. nobile*, respectively. The SSR analysis also revealed types of nucleotide repeats (mono-, di- and tetra-) in the cp DNA of *Dendrobium* species, which can serve as potential barcode markers for species discrimination (Fig. 3.

**Figure:**
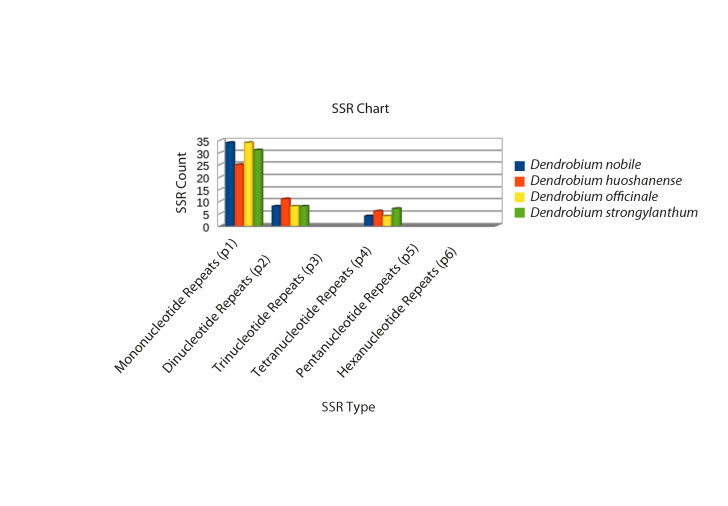
**Fig. 3: SSR analysis of the cp DNA from four *Dendrobium* species.** Three types of SSRs (mono-, di- and tetranucleotide repeats) were revealed in the cp DNA of *D. nobile*, *D. officinale*, *D. huoshanense* and *D. strongylanthum*. Different colour codes represent SSRs in different *Dendrobium* species.

**Table:** **Table 1.** Sequence information for the cp DNA of *Dendrobium nobile* isolates from India and China.

Information	LC011413	KX377961
Sequence type	DNA	DNA
Length	150,793bp circular	152,018bp circular
Organism	Dendrobium nobile	Dendrobium nobile
Name	LC011413	KX377961
Description	Dendrobium nobile chloroplast DNA, complete genome.	Dendrobium nobile chloroplast, complete genome.
Modification Date	02-AUG-2016	10-AUG-2016
Weight (single-stranded)	46.552 MDa	46.932 MDa
Weight (double-stranded)	93.156 MDa	93.912 MDa

**Table:** **Table 2:** Nucleotide counts for the cp DNA of *Dendrobium nobile* isolates from India and China.

Nucleotide	LC011413	KX377961
Adenine (A)	46,152	46,576
Cytosine (C)	28,739	28,853
Guanine (G)	27,891	28,039
Thymine (T)	48,009	48,381
Purine (R)	0	31
Pyrimidine (Y)	0	20
Adenine or cytosine (M)	0	56
Guanine or thymine (K)	0	31
Cytosine or guanine (S)	0	3
Adenine or thymine (W)	0	28
Not adenine (B)	0	0
Not cytosine (D)	0	0
Not guanine (H)	0	0
Not thymine (V)	0	0
Any nucleotide (N)	2	0
C + G	56,630	56,892
A + T	94,161	94,957

**Table:** **Table 3:** Annotated genomic features for the cp DNA of *Dendrobium nobile* isolates from India and China.

Feature type	LC011413	KX377961
CDS	73	79
Exon	0	22
Gene	127	132
Misc. feature	9	2
Repeat region	2	2
Source	1	1
rRNA	8	8
tRNA	39	38

**Table:** **Table 4:** Nucleotide frequencies in the cp DNA of Indian and Chinese *Dendrobium nobile* isolates.

Nucleotide	LC011413	KX377961
Adenine (A)	0.306	0.306
Cytosine (C)	0.191	0.190
Guanine (G)	0.185	0.184
Thymine (T)	0.318	0.318
Purine (R)	0.000	0.000
Pyrimidine (Y)	0.000	0.000
Adenine or cytosine (M)	0.000	0.000
C + G	0.376	0.374
A + T	0.624	0.625

**Table:** **Table 5:** Codon usage frequency in the cp DNA of *Dendrobium nobile* isolates from India and China.

Codon	LC011413	KX377961
AAA	0.04	0.04
AAC	0.01	0.01
AAG	0.02	0.02
AAT	0.04	0.04
ACA	0.02	0.02
ACC	0.01	0.01
ACG	0.01	0.01
ACT	0.02	0.02
AGA	0.02	0.02
AGC	0.00	0.00
AGG	0.01	0.01
AGT	0.02	0.02
ATA	0.02	0.02
ATC	0.02	0.02
ATG	0.02	0.02
ATT	0.04	0.04
CAA	0.03	0.03
CAC	0.01	0.01
CAG	0.01	0.01
CAT	0.02	0.02
CCA	0.01	0.01
CCC	0.01	0.01
CCG	0.00	0.00
CCT	0.02	0.02
CGA	0.01	0.01
CGC	0.00	0.00
CGG	0.00	0.00
CGT	0.01	0.01
CTA	0.01	0.02
CTC	0.01	0.01
CTG	0.01	0.01
CTT	0.02	0.02
GAA	0.04	0.04
GAC	0.01	0.01
GAG	0.01	0.01
GAT	0.03	0.03
GCA	0.02	0.02
GCC	0.01	0.01
GCG	0.01	0.01
GCT	0.02	0.02
GGA	0.03	0.03
GGC	0.01	0.01
GGG	0.01	0.01
GGT	0.02	0.02
GTA	0.02	0.02
GTC	0.01	0.01
GTG	0.01	0.01
GTT	0.02	0.02
TAA	0.00	0.00
TAC	0.01	0.01
TAG	0.00	0.00
TAT	0.03	0.03
TCA	0.02	0.02
TCC	0.01	0.01
TCG	0.01	0.01
TCT	0.02	0.02
TGA	0.00	0.00
TGC	0.00	0.00
TGG	0.02	0.02
TGT	0.01	0.01
TTA	0.03	0.03
TTC	0.02	0.02
TTG	0.02	0.02
TTT	0.03	0.03

## Conclusion

Chloroplast genome sequences serve as valuable assets in herbal medicine. As many medicinal plants are highly endangered and rare in nature, little information is available to confirm their identity. Bio-barcodes derived from chloroplast genomes are quite useful for identifying species varieties and resources. Functional and structural annotations of gene content, gene organization, and chloroplast genome sequences have been used as important markers in systematic research. This report determined the complete chloroplast genome sequence of *D. nobile* from Northeast India. We found structural similarities among the taxa of different subfamilies of Orchidaceae and also identified differences in IR/SSC junctions and ndh genes from other orchid plastid genomes. Our phylogenetic analyses reveal that *D. nobile* is most closely related to *D. officinale* and *D. pendulum*. In addition, relationships among subfamilies in the family Orchidaceae were resolved in the present study. The highly divergent genes in the cp genomes identified in this study can be used as potential molecular markers in phylogenetic analyses. In summary, the results of this study will further our understanding of the evolution, molecular biology and genetic improvement of the medicinal orchid *D. nobile*.

## Data Availability

The entire chloroplast sequence is available from NCBI GenBank with the accession number KX377961 (https://www.ncbi.nlm.nih.gov/nuccore/KX377961).

## Competing Interests

The authors have declared that no competing interests exist.

## Corresponding Authors

Devendra Kumar Biswal, Bioinformatics Centre, North-Eastern Hill University, Shillong- 793022, Meghalaya, India

Email: devbioinfo@gmail.com

Pramod Tandon, Biotech Park, Kursi Road, Lucknow- 226021, Uttar Pradesh, India

Email: profptandon@gmail.com
